# Validation of an Analytical Method for Determination of 13 priority polycyclic aromatic hydrocarbons in mineral water using dispersive liquid-liquid microextraction and GC-MS

**Published:** 2016

**Authors:** Ramezan Sadeghi, Farzad Kobarfard, Hassan Yazdanpanah, Samira Eslamizad, Mitra Bayat

**Affiliations:** a *Department of Toxicology and Pharmacology, School of Pharmacy, Shahid Beheshti University of Medical Sciences, Tehran, Iran. *; b *Department of Environmental Health Engineering, School of Health, Shahrekord University of Medical sciences, Shahrekord, Iran.*; c *Department of Medicinal Chemistry, School of Pharmacy, Shahid Beheshti University of Medical Sciences, Tehran, Iran. *; d *Phytochemistry Research Center, Shahid Beheshti University of Medical Sciences, Tehran, Iran. *; e *Food Safety Research Center, Shahid Beheshti University of Medical Sciences, Tehran, Iran.*

**Keywords:** GC-MS, Polycyclic aromatic hydrocarbons (PAHs), mineral water, Dispersive Liquid-Liquid Microextraction (DLLME), Iran

## Abstract

Dispersive liquid-liquid microextraction (DLLME) combined with gas chromatography–mass spectrometry (GC–MS) was used for the extraction and determination of 13 polycyclic aromatic hydrocarbons (PAHs) in mineral water samples. In this procedure, the suitable combination of extraction solvent (500 µL chloroform) and disperser solvent (1000 µL acetone) were quickly injected into the water sample (10.00 mL) by Hamilton syringe. After centrifugation, 500 µL of the lower organic phase was dried under a gentle stream of nitrogen, re-dissolved in chloroform and injected into GC-MS. Chloroform and acetone were found to be the best extraction and disperser solvent, respectively. Validation of the method was performed using spiked calibration curves. The enrichment factor ranged from 93 to 129 and the recovery ranged from 71 to 90%. The linear ranges for all the PAHs were 0.10-2.80 ngmL^-1^. The relative standard deviations (RSDs) of PAHs in water by using anthracene-d_10 _as internal standard, were in the range of 4-11% for most of the analytes (n = 3). Limit of detection (LOD) for different PAHs were between 0.03 and 0.1 ngmL^-1^. The method was successfully applied for the analysis of PAHs in mineral water samples collected from Tehran.

## Introduction

Polycyclic aromatic hydrocarbons (PAHs) are a group of various cyclic organic compounds with two or more fused aromatic rings in their structures (1). PAHs are principally byproducts of incomplete burning of organic substances occurring naturally or the result of human intervention such as jungle fires, domestic and industrial heating appliances, factories and power plants to generate electricity, transportation industry, cooking, volcanic activities and so on (2-5). Therefore, due to such extensive sources, they have been noticed in water, air, soil, agriculture products and nearly everywhere in the environment (3, 6). The possible sources of PAHs in water may be listed as below: Atmospheric deposition (via wet and dry particle deposition and gross gas absorption), wastewater treatment plant discharges, tributaries, storm water runoff, oil spills, ground water discharges from underground water and runoff of PAHs from contaminated sites (7). After the arrival of PAHs to the atmosphere, they are transferred into water by direct surface contact or as a result of precipitation (8). Occurrence of PAHsinwater resources, including drinking water have been reported indifferent parts of the world (8). WHO reported that water is a very significant source of PAHs, and in drinking water, fluoranthene, phenanthrene, pyrene and anthracene were usually detected (9, 10). Among the routes of exposure of general public such as inhalation of ambient and indoor air, dermal absorption, and/or dietary intake, drinking water is also important (11, 12).

Naphthalene is the most abundant PAH in water and the U.S. Department of Health and Human Services has concluded that it is reasonably anticipated to be a human carcinogen. However, PAHs show an extensive range of toxicities. For example, according to the international agency for research on cancer (IARC) classification system, benzo[a]pyrene (BaP) as one of the most known is classified in group 1 and some of them are also categorized as group 2A or 2B carcinogens (13, 14). Due to their recognized and doubted carcinogenicity and/or mutagenicity, many of these compounds form part of public health concern (15). It has not been possible to define a threshold level for PAHs, below which risk would be insignificant and therefore a tolerable daily intake could not be set for these compounds. As a result, it was suggested that exposures to them should be as low as reasonably achievable (16). The international organizations, i.e. World Health organization (WHO), Environmental Protection Agency (EPA) and European Community (EC), have recommended the continual detection and quantification of these compounds in drinking waters (17) and established the maximum residue levels (MRLs) for benzo[a]pyrene (BaP) as the most well-known component of this group (18).

Due to low solubility, the concentration of organic pollutants, especially PAHs in water is extremely low (A). Therefore, despite the use of exceedingly sensitive analytical devices for the analysis of samples the results will not have enough accuracy and precision (19). Consequently, it is essential to develop and validate an extraction method and preconcentration procedures for these compounds prior to their instrumental analysis (19). The sample preparation method, must have high enough efficiency in separation and concentration of the trace analytes from the matrix and is well-matched with analytical device (20). 

In order to separate, clean up and concentrate PAHs from water samples, many methods including liquid–liquid extraction (LLE) (21-28), solid-phase extraction (SPE) (25, 29-32), liquid phase microextraction (LPME) (33-36), dispersive liquid–liquid microextraction (DLLME) (37-40), micelle-mediated preconcentration (MMP) (41), hollow fiber-based LPME technique (HF-LPME) (42). single-drop microextraction (SDME) (43), Dispersive Liquid-Liquid Microextraction (DLLME) based on the Solidification of Floating Organic Drop (SFO), ultrasound-assisted emulsification microextraction (USAEME) (44), and acid-induced cloud point extraction (ACPE) (45), hollow fiber liquid–liquid microextraction (HF-LLME) are used (37). Due to time consuming and high cost of LLE and SPE methods, the miniaturized extraction procedures such as DLLME have mostly been considered (6, 24, 37). DLLME method has been presented by Rezaee *et al*. in 2006. In this procedure a suitable combination of dispersive and extraction solvents (a polar water miscible, a high-density solvent, respectively) is pushed into an aqueous sample using a microsyringe. After forming a cloudy solution and centrifugation, the content of the lower phase can be analyzed by an appropriate analytical method (38). DLLME procedure has many advantages such as low cost and therefore, has been used by many researchers for aqueous sample preparation (46). In this study, parameters affecting DLLME procedure and GC-MS techniques were examined and optimized to determine 13 priority PAHs in real mineral water samples collected from Tehran market. In the European Union, bottled water may be called mineral water when it is bottled at the source and has undergone no or minimal treatment. The U.S. Food and Drug Administration (FDA) classifies mineral water as water containing at least 250 parts per million total dissolved solid (TDS), originating from a geologically and physically protected underground water source. In many places, however, the term “mineral water” is colloquially used to mean any bottled water as opposed to tap water. 

## Experimental

*Reagents and materials*

Naphthalene (Naph.), acenaphthene (Ace.), flourene (Fl.), phenanthrene (Phen.), anthracene (Ant.), anthracene-d10 (Ant.d10), pyrene (Pyr.), benzo[a]anthracene (B[a]A), chrysene (Chy.), benzo[b]fluoranthene (B[b]F), benzo[k]fluoranthene (B[k]F), benzo[a]pyrene (B[a]P), dibenz[a,h]anthracene (D[ah]A) and benzo[ghi]perylene (B[ghi]P) with purity higher than 98% were purchased from Sigma-Aldrich/Fluka/ Supelco (Germany). HPLC grade isooctane, toluene, acetonitrile, dichloromethane, tetrachloroethylene, n-hexane, ethanol, 2-propanol, methanol, acetone and chloroform were obtained from Chem-lab Belgium. Ultrapure water was obtained from a Milli-Q plus ultra-pure water system (Millipore, Molsheim, France). Mineral water samples were collected from Tehran and used without any prior treatment. Stock standard solutions of PAHs were individually prepared by dissolving 10 mg of each in 10 mL toluene of HPLC grade (Merck, Germany). 

*Calibration standards*

Individual stock standard solutions (1 mgmL^-1^) of the PAHs were prepared in toluene. All the solutions were transferred to amber glass vials and stored at 4°C. They were kept for 30 min. at ambient temperature prior to their use. A mixed intermediate standard solution at a concentration of 100 ngmL^-1^ was prepared via appropriate dilution of the stock solutions in methanol. This solution was used as a spiking solution for validation experiments. Spiked calibration standards at concentration levels of 0.35, 0.7, 1.4, 2.8, and 5.6 ngmL^-1 ^were prepared by addition of 35 μL, 70 μL, 140 μL, 280 μL and 560 μL of mixed standard stock solution to 10 mL of blank water samples in each case.

*GC–MS analysis*

Analyses were carried out by using a 7000 Agilent triple Quadrupole MS system coupled with a 7890A GC, equipped with a split/splitless injection port, an autosampler model Agilent 7693, and electronic ionization. A HP-5MS 5% Phenyl Methyl Silox, Agilent 19091s-433 capillary column was used (30 m × 0.25 mm I.D. and 0.25 μm film thickness). Helium with a purity of 99.99% and a flow rate of 1 mLmin^-1 ^was used as carrier gas. 

*Method validation*

Method efficiency is a very important parameter which should be assessed by all testing laboratories to guarantee the validity of routine analysis (47). In the present study, the spiked calibration curves (five points) for all the analytes were attained by plotting the ratio of peak area of each compound to that of internal standard against the concentration of the corresponding analyte. The graphs were constructed using triplicate analysis over the concentration range of 0.1-2.8 ngmL^-1^. Recovery and repeatability were determined at concentration levels of 0.4, 1 and 2.5 ngmL^-1^. Each concentration level was repeated 3 times per day, and this was performed for three consecutive days. Internal standard (anthracene-d10) was used at a concentration equal to 0.7 ngmL^-1^. Blank sample (n = 3) was analyzed along with other samples every day. For each compound, the mass fragment (m/z) with the highest intensity was selected as quantifier ion and its peak area at different concentration levels was used to construct the calibration curve. Limits of detection (LODs) and Limits of quantification (LOQs) were calculated based on the signal-to-noise ratio of equal to 3 and 10, respectively. The validation parameters were compared to EU provision No. 836/2011 (47).

*DLLME procedure*

Ten milliliter ultrapure water was placed in a 15 mL screw cap glass test tube and spiked with PAHs and anthracene-d10 (as internal standard) at concentrations 1 and 0.5 µgmL-1, respectively. A mixture of 500 µL chloroform and 1000 µL acetone (as extraction and disperser solvent, respectively) was quickly injected into the sample solution in three portions, with a 500 µL Hamilton syringe. A cloudy solution was formed after adding the mixture of extraction and disperser solvent in the test tube. The test tube containing ultrapure water, extraction and disperser solvent was centrifuged (Hettich Zentrifugen Universas 320R) for 3 min at 2500 rpm. The upper layer was discarded and 500 µL of the lower phase (extraction solvent) was removed by a Hamilton syringe and transferred to 1.5 mL amber glass vial. The content of the vial was dried under a gentle stream of nitrogen at room temperature and re-dissolved in 70 µL chloroform. The vial was placed in auto sampler and 2 µL of the contents was injected into the gas chromatograph in splitless mode. The approximate volume of the sedimented phase was about 550 µL.

*Calculation of enrichment factor and extraction recovery*

The enrichment factor (EF) is defined as the ratio between the analyte concentration in the sedimented phase (C_sed._) and the spiking level (C_0_) (38). The C_sed._was obtained from calibration graph of direct injection of PAHs standard solution in methanol at the range of 0.25-5 mgL^-1^.

The extraction recovery (ER) is defined as the percentage of the total analyte amount in the sedimentedphase (C_sed. _× V_sed._) to total analyte amount in aqueous solution (V_aq _× C_0_) (38).

## Results and Discussion

*Gas chromatography mass spectrometry determination*

Analysis was accomplished in the SIM mode based on the use of one target as quantification ion and two confirmation ions. PAHs were recognized according to their retention times, target and confirmation ions (Table 1). The quantification was based on the peak area ratio of the targets to that of internal standard.In this study, the injector temperature was retained at 280°C and injection was performed in the splitless mode. The initial oven temperature was maintained at 60°C for 0.5 min, increased to 230°C at a ramp rate of 3°C min^−1^ and kept for 0.5 min, then increased to 290°C at 5°C min−1 and hold for 10 min at the final temperature. Data acquisition was delayed for 12 min. The ionization was performed in ion source with electron impact mode (70ev). The ion source and triple quadrupole mass analyser temperature were kept at 230 and 280°C, respectively. A mass range of m/z 50-500 was scanned to find the retention time and diagnostic ions (quntification and confirmation ions) of the analytes. Retention time and mass spectrum of each of the standards were used to identify and confirm them. For quantitative determination, PAH standards and samples were analyzed in selected ion monitoring (SIM) mode. The retention time, diagnostic ions and quantification ion for each analyte are presented in Table1. 

**Table 1:** Retention time and selected diagnostic ions of the studied analytes.

Retention time (min)	Diagnostic ions (m/z)		PAHs
	Quantification ion	Confirmation ions m/z (%)	
18.203	128	127(44.8), 129(33.5), 102(22.5)	Naphthalene
30.846	153	154(93.2), 152(53.1), 151(24.6)	Acenaphthene
34.790	166	165(94.0), 167(17.6), 139(10.5)	Fluorene
42.151	178	179(18.8), 176(23.7), 152(13.5)	Phenanthrene
42.292	188	189( 16.7), 187(13.9), 160(11.1)	Anthracene-d10
42.392	178	179(19.2), 176(24.5), 152(12.2)	Anthracene
52.786	202	203(20.3), 200(22.1), 101(21.8)	Pyrene
59.971	228	226(28.4), 229(22.6), 227(10.7)	Benz[a]anthracene
60.217	228	226(31.3), 229(22.6), 113(23.9)	Chrysene
68.106	252	253(68.6), 250(28.1), 126(55.6)	Benzo[b]fluoranthene
68.176	252	253(24.4), 250(23.5), 126(23.9)	Benzo[k]fluoranthene
68.634	252	253(27.4), 250(24.1), 126(21.1)	Benzo [a]pyrene
75.396	278	276(29.3), 138(32.8), 139(32.5)	Dibenz[a,h]anthracene
76.699	276	277(25.1), 274(23.1), 138(43.0)	Benzo[g,h,i]perylene

*Method validation*

According to the calibration graphs, the linearity for Naphthalene, Acenaphthene, Flourene and for Phenanthrene, Anthracene, Pyrene, Benz[a]anthracene, Chrysene, Benzo[b]fluoranthene, Benzo[k]fluoranthene, Benzo[a]pyrene, Dibenz[a,h]anthracene, Benzo[ghi]perylene were observed over the concentration range of 0.1-2.8 and 0.35-2.8 ngmL^-1^, respectively. The coefficients of determination (r^2^) were between 0.983 and 0.999 for all PAHs. It shows that the extraction process and analytical method after validation have enough efficiency for the determination of PAHs at trace levels. In the present study, we used spiked calibration standard approach to overcome the problems caused by the matrix. In this approach, calibration standards are prepared by the addition of standard solution to blank water samples that are subjected to the same sample preparation procedure which is intended to be used for unknown samples. In this way, the standard sample matrices will have the same composition as the unknown samples and therefore the effect of matrix is reflected in both standards and unknown samples. The calibration curve is constructed using these spiked calibration standards and it is easily used to calculate the concentration of analyte (s) in unknown sample without being concerned about the matrix effects. The developed method has the advantage of using spiked calibration curves that minimize the matrix interferences.

Limits of detection (LODs) and Limits of quantification (LOQs) were calculated based on the signal-to-noise ratio of equal to 3 and 10, respectively. Recovery, repeatability, RSD%, R^2^, LODs, LOQs and HORRAT of the method under optimized conditions are summarized in tables 2 and 3. As shown in these tables, all of the validation parameters examined were in compliance with EU provision No. 836/2011 (47). Therefore, the attained validation parameters are acceptable and the optimized and validated method can be used to analyze the real samples. Hence, the optimized method were applied to analyze 50 mineral water samples which were collected from Tehran market.

**Table 2:** Accuracy and precision of the DLLME procedure and GC-MS method in determination of 13 PAHs in ultrapure water (n = 3)^a^.

HORRAT^b^	AverageCV(%)	Average Recovery(%)	Spiking level (ngmL^-1^)						PAHs
			2.5		1.0		0.4		
			CV (%)	Recovery (%)	CV (%)	Recovery (%)	CV (%)	Recovery (%)	
0.5	11	71	12	73	6	87	16	52	Naphthalene
0.4	9	81	12	77	6	94	8	72	Acenaphthenen
0.2	5	81	7	80	4	95	4	68	Flourene
0.2	5	79	10	80	3	96	4	62	Phenanthrene
0.2	5	84	4	82	5	97	5	74	Anthracene
0.5	11	65	7	59	8	72	18	65	Pyrene
0.4	8	85	5	66	9	85	11	103	Benzo[a]anthracene
0.3	6	72	4	60	11	78	4	79	chrysene
0.4	8	84	6	55	10	82	6	116	Benzo[b]fluoranthene
0.2	4	88	6	84	3	94	5	86	Benzo[k]fluoranthene
0.4	9	83	8	58	11	82	9	109	Benzo[a]pyrene
0.8	17	90	17	63	20	104	14	102	Dibenz[a,h]anthracene
0.5	10	71	6	55	10	77	15	80	Benzo[ghi]perylene

a _Extraction conditions: extraction method, DLLME procedure; ulrapure water sample volume, 10 mL; spiking level, 0.4-2.5 ngmL-1; disperser solvent (acetone) volume, 1000 µL; extraction solvent (chloroform) volume, 500 µL; internal standard (anthracene–d10), 0.7 ngmL-1; centrifugation time and rpm, 3min, 2500, room temperature._

b _HORRAT, the Horwitz Ratio._

**Table 3:** Figures of merit for the DLLME procedure and GC-MS method in determination of 13 PAHs in ultrapure water^a^

Regression equation	LR^e^ (ngmL^-1^)	EF^d^	r^2^	LOD^c^ (ngmL^-1^)	LOQ^b^ (ngmL^-1^)	PAHs
y= 2.5070 x +0.6869	0.10-2.80	101.40	0.992	0.03	0.10	Naphthalene
y=1.3637 x +0.0954	0.10-2.80	115.78	0.995	0.03	0.10	Acenaphthene
y=1.3686 x +0.1663	0.10-2.80	115.71	0.997	0.03	0.10	Flourene
y=3.6569 x +0.7157	0.35-2.80	112.86	0.995	0.10	0.35	Phenanthrene
y=3.1554 x +0.3356	0.35-2.80	120.00	0.996	0.10	0.35	Anthracene
y=1.4566 x +0.0772	0.35-2.80	92.86	0.996	0.10	0.35	Pyrene
y=0.7353 x -0.0828	0.35-2.80	121.43	0.987	0.10	0.35	Benzo[a]anthracene
y=0.8012 x +0.0014	0.35-2.80	102.86	0.995	0.10	0.35	chrysene
y=0.5118 x -0.1270	0.35-2.80	120.00	0.983	0.10	0.35	Benzo[b]fluoranthene
y=0.6653 x -0.0660	0.35-2.80	125.71	0.996	0.10	0.35	Benzo[k]fluoranthene
y=0.3071 x -0.0501	0.35-2.80	118.57	0.993	0.10	0.35	Benzo[a]pyrene
y=0.0389 x +0.0038	0.35-2.80	128.57	0.999	0.10	0.35	Dibenz[a,h]anthracene
y=0.0705 x -0.0034	0.35-2.80	101.43	0.999	0.10	0.35	Benzo[ghi]perylene

a _Extraction conditions: extraction method, DLLME procedure; ulrapure water sample volume, 10 mL; spiking level, 0.1-2.8 ngmL-1; disperser solvent (acetone) volume, 1000 µL; extraction solvent (chloroform) volume, 500 µL; internal standard (anthracene–d10), 0.7 ngmL-1; centrifugation time and rpm, 3min, 2500, room temperature._

b _LOQ, limit of quantification for a S/N = 9._

c _LOD, limit of detection for a S/N = 3._

d _EF, enrichment factor._

e _LR, linear range._

*Dispersive liquid-liquid microextraction optimization*

The recovery rate is an important indicator at the extraction process. It was usually used to evaluate the efficiency of the DLLME procedure. There are many parameters that affect the recovery. In the present study, the effect of different factors such as the type and volume of extraction and disperser solvent, rpm and time of centrifuge on the recovery were evaluated and optimized. To evaluate the effect of these parameters on the recovery, we used 10.00 mL of ultrapure water containing 1.00 ngmL^-1^ of each PAH and 0.70 ngmL^-1^ of antracene-d10 as internal standard. All the optimization trials were performed three times.

*The kind of extraction solvent*

**Figure 1:**
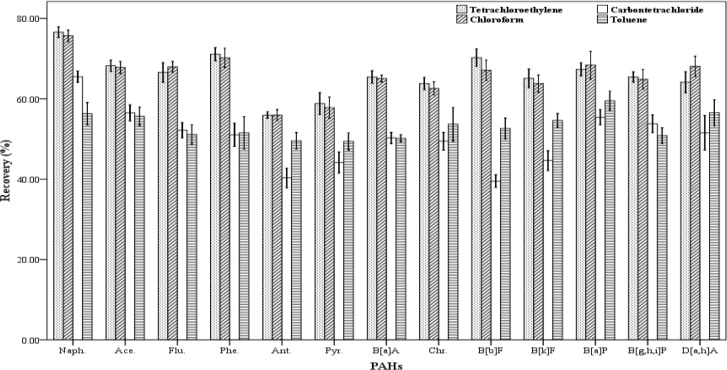
Effect of the kind of extractant (n = 4) on the recovery of PAHs. Extraction conditions

 In order to have efficient extraction, the selection of a suitable extraction solvent is essential. The extraction solvent must have some characteristics including: low solubility in water, low volatility, High affinity to extract the desired compounds and the ability to form a clear and detectible peak in the chromatogram (18). In this study, different organic solvents were used as extraction solvent with higher or lower density than water. The solvents including tetrachloroethylene, toluene, carbon tetrachloride, chloroform and n-hexane were examined and their ability to extract the analytes were assessed (Figure 1). In order to evaluate the extraction efficiency of each of the organic solvents, three spiked samples were prepared. The extraction and centrifugation processes were fulfilled according to the stated method. But instead of 500 µL of each extraction solvent, 250 µL was used. The recovery values for the 13 studied PAHs are shown in Figure 1. The results indicated that chloroform and tetra chloroethylene have higher extraction efficiency than the others. The extraction efficiencies attained for chloroform and tetra chloroethylene are similar and there were not any significant differences in recovery values between them. But a number of compounds did not result in normal peaks when tetra chloroethylene was used to extract them. Therefore, chloroform was chosen as the optimum extraction solvent. 

extraction method, DLLME procedure, ultrapure water sample volume, 10 mL, spiking level, 2.8 ngmL^-1^; disperser solvent (acetone) volume, 1000 µL; extraction solvent volume, 500 µL; centrifugation, 3 min., 2500 rpm, room temperature.

*Extraction solvent volume*

**Figure 2:**
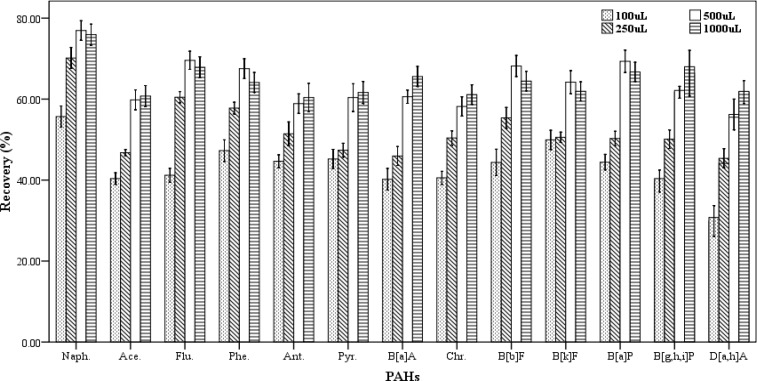
Effect of the extraction solvent volume on the recovery of PAHs. Extraction conditions: extraction method, DLLME procedure, ultrapure water sample volume, 10 mL; spiking level, 2.8 ngmL^-1^; disperser solvent volume, 1000 µL; extraction solvent, chloroform; centrifugation, 3 min., 2500 rpm, room temperature

To evaluate the effect of extraction solvent volume on the recovery values of the studied analytes, different volumes of chloroform (100, 250, 500 and 1000 µL) were examined. In this study, three spiked samples were prepared for each of the volumes of the extracting solvent. At all the experiments a constant volume of 1000 µL of the dispersive solvent was applied. The extraction and centrifugation processes were done according to procedure described in section 2.3. As shown in Figure 2, the recovery values of PAHs increase gradually with rising the volume of the extracting solvent in the range of 100-500 µL. Figure 2 shows that the volumes of 500 and 1000 µL of the extraction solvent have the highest extraction efficiencies but there is not any significant differences in extraction efficiency between them. The results had no repeatability in case of the volumes less than 100 µL. Therefore, the volume of 500 µL of chloroform was chosen as the best volume of extraction solvent. 

*The kind of disperser solvent*

**Figure 3:**
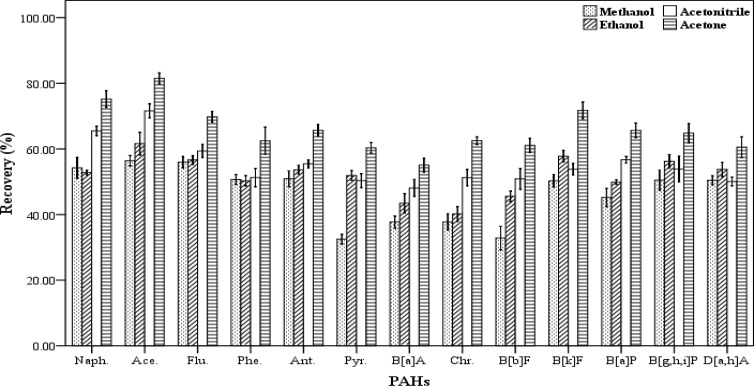
Effect of different disperser solvent volume (n = 4) on the recovery of PAHs. Extraction conditions: extraction method, DLLME procedure, ultrapure water sample volume, 10 mL; spiking level, 2.8 ngmL^-1^; disperser solvent volume, 1000 µL; extraction solvent volume (chloroform), 500 µL; centrifugation, 3 min., 2500 rpm, room temperature

The selection of an appropriate disperser solvent is very important for the efficient extraction. The disperser solvent must have some characteristics including: the ability to be distributed in aqueous samples in order to form very small droplets and increase the surface area in contact between the analytes and the extracting solvent and solubility in the aqueous samples and the organic extraction solvents. In addition, the compound should also be not expensive and highly toxic. Therefore, the ability to form a cloudy solution or an effective emulsion depends significantly on the amount and type of the disperser solvents. In this work, acetone, acetonitrile, methanol and ethanol were tested and their influence on the recovery values were evaluated (Figure 3). For this purpose, five spiked samples were supplied for each of disperser solvent. Then each of them was extracted by using a combination of 500 µL of chloroform as the extracting solvent and 1000 µL of each of the mentioned disperser solvents. The extraction and centrifugation processes were accomplished based on the aforesaid procedure in section 2.3. All the lower phase was collected and dried, then re-dissolved in 70 µL of chloroform. The results of these experiments are presented in Figure 3. Maximum recovery values obtained for all 13 PAHs when acetone was used as disperser solvent compared to the others. Therefore, acetone was selected and used as disperser solvent in this study.

*Dispersive solvent volume*

**Figure 4:**
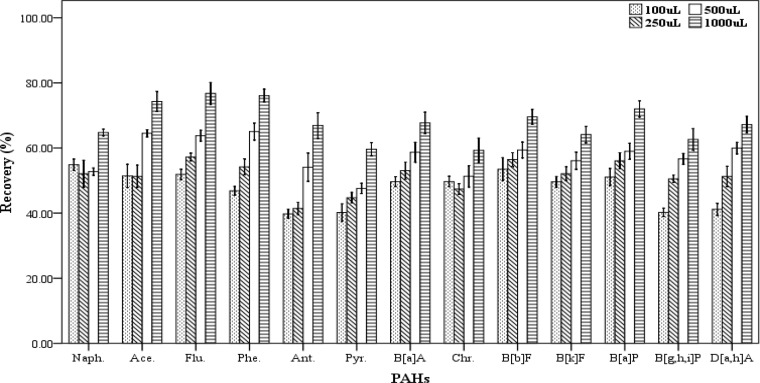
Effect of the disperser solvent volume (n = 4) on the recovery of PAHs. Extraction conditions: extraction method, DLLME procedure, ultrapure water sample volume, 10 mL; spiking level, 2.8 ngmL^-1^; disperser solvent, acetone, extraction solvent volume, 500 µL; centrifugation, 3 min., 2500 rpm, room temperature

In DLLME procedure, the dispersive solvent volume is a critical parameter and its effect should be determined on the efficiency of extraction. For examining the effect of this factor on the recovery values of the 13 PAHs, five spiked samples were prepared for each of the dispersive solvent volume. In this work, the different volumes (100, 250, 500 and 1000 µL) of acetone as dispersive solvent containing 500 µL chloroform as the extracting solvent were applied for extraction. The extraction and centrifugation processes were accomplished based on the aforementioned technique in Section 2.3. The settled phase was collected completely and dried, then re-dissolved in 70 µL of chloroform. As shown in Figure 4, increasing the dispersive solvent volume results gradually in increased recovery values of PAHs. The highest efficiency of extraction was achieved when 1000 µL of acetone was used. Then, a mixture of the extracting and dispersve solvents with volumes of 500 and 1000 µL, respectively were selected as the optimum volumes for further trials.

*Centrifugation time*

**Figure 5:**
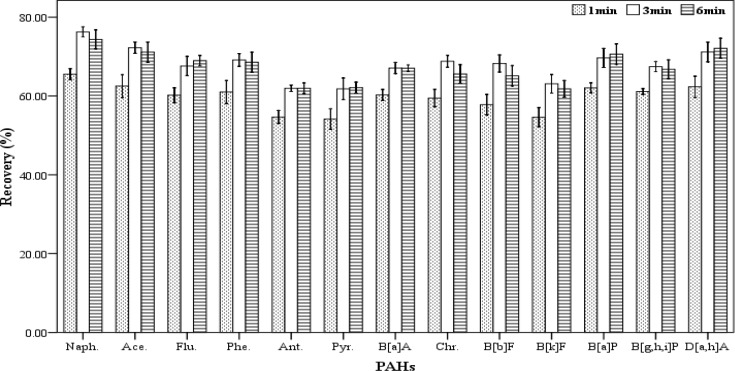
Effect of centrifugation time (n = 3) on the recovery of PAHs. Extraction conditions: extraction method, DLLME procedure; ultrapure water sample volume, 10 mL; spiking level, 2.8 ngmL^-1^; disperser solvent (acetone) volume, 1000 µL; extraction solvent (chloroform) volume, 500 µL; centrifugation rpm, 2500, room temperature

In order to assess the effect of centrifugation time on the extracting efficiency, a range of centrifugation times (1, 3 and 6 min) were tried. For each of the mentioned times, five spiked samples were prepared. For extraction, a mixture containing 500 µL chloroform and 1000 µL acetone were used as the extracting and disperser solvents, respectively. The extraction process was accomplished according to the described procedure. All of the experiments were performed at 5000 rpm. The results (recovery values) are shown in Figure 5. According to the results, the centrifugation times 3 and 6 minutes have the highest effect on the amount of recovery but there was not a significant difference between them. Therefore, 3 min was applied as the optimum centrifugation time for further tests.

*Analysis of real samples*

Khalili Zanjani MR, *et al.* (26) and Rezaee M, *et al.* (38) have reported the use of DLLME method along with GC-FID for the analysis of polycyclic aromatic hydrocarbons.

Khalili Zanjani MR, *et al*. have used 1-undecanol as the extraction solvent while Rezaee M, *et al*., have used tetra chloroethylene for extraction. 

In the present study, chloroform was used as the extraction solvent and mass detector was used coupled to the gas chromatograph.

Satisfactory figures of merit were obtained. The optimized and validated procedure was used to determine PAHs in 50 real water samples (bottled mineral water). No treatment was conducted such as filtration on the samples, prior to extraction process. Extraction process, centrifugation and analysis were accomplished according to the described procedure. A blank and three spiked ultrapure water at concentration of 1 ngmL^-1^ were used beside the real water samples, per working day. The results of the samples analysis show that the concentrations of PAHs in the real samples (mineral water samples) were lower than the LOQ in all the samples. There are only few published papers for determination of PAH in mineral water. In one of these studies, Humood F. Al-Mudhafa *et al*. (48) analyzed twenty-five bottled water brands for extractable semivolatiles (SVs), including PAHs, listed in the US-EPA 525.2 method; and found that all samples were free of SVs contamination. Our results are in agreement with the above mentioned study. 

But in some studies, some contamination have been found in mineral water. For example, In one of these studies, Ma Teresa Pena *et al.* (49) determined eighteen PAHs in drinking water samples (tap, bottled, fountain, well) and reported sum of their concentration between 127.8 ngL^−^^1 ^and 413.2 ngL^−^^1^. In another study, Albert Guart *et al*. (50) detected PAHs in 18 out of 77 bottled water samples at levels between 0.005 and 0.202 μgL^-1^. They found that the most detected compound was naphthalene, which was detected in 16 samples at concentrations of 0.005-0.202 μgL^-1^. Also, CuneytGuler, (51) found that a significant number of bottled waters in Turkey contained some elements (*e.g*. sodium, chloride, sulfide, fluoride, PAHs and several heavy metals) above the maximum concentration allowed for bottled waters by the Turkish legislation. 

Considering the few studies regarding PAHs contamination in drinking water in Iran, to get a clear picture of contamination of drinking water, comprehensive monitoring of PAHs in water in different provinces and seasons are suggested.

## Conclusions

In the present study, dispersive liquid-liquid microextraction (DLLME) procedure coupled with gas chromatography mass spectreometry was used for determination of trace amounts of thirteen polycyclic aromatic hydrocarbons in water samples. After optimization of the parameters affecting the extraction and analysis efficiency, the identification and quantification of the PAHs were carried out by applying the mentioned techniques for the analysis of mineral water samples. The results showed that these two techniques in combination, result in a satisfactory recovery and precision. No contamination of mineral water samples with PAHs were found among the samples. However, considering the high risk of contamination with PAHs in Iran, a comprehensive survey for PAHs in water is recommended.

## References

[1] Ma J, Xiao R, Li J, Yu J, Zhang Y, Chen L (2010). Determination of 16 polycyclic aromatic hydrocarbons in environmental water samples by solid-phase extraction using multi-walled carbon nanotubes as adsorbent coupled with gas chromatography–mass spectrometry. J Chromatogr A.

[2] Sanchesa S, Leitãob C, Penetrac A, Cardosoc VV, Ferreirac E, Benolielc MJ, Barreto Crespoa MT, Pereira VJ (2011). Direct photolysis of polycyclic aromatic hydrocarbons in drinking water sources. J Hazardous Materials.

[3] Díaz-Moroles NE, Garza-Ulloa HJ, Castro-Ríos R, Ramírez-Villarrea EG, Barbarín-Castillo JM, de la Luz Salazar-Cavazo M, Waksman-de Torres N (2007). A comparison of the performance of two chromatographic and three extraction techniques for the analysis of PAHs in sources of drinking water. J Chromatogr Sci.

[4] Zhang L, Dong L, Ren L, Shi S, Zhou L, Zhang T, Huang Y (2012). Concentration and source identification of polycyclic aromatic hydrocarbons and phthalic acid esters in the surface water of the Yangtze River Delta. China J Environ Sci.

[5] Ozcan S, Tor A, Aydin ME (2010). Determination of polycyclic aromatic hydrocarbons in waters by ultrasound-assisted emulsification-microextraction and gas chromatography–mass spectrometry. Anal Chim Acta.

[6] Khalili-Fard V, Ghanemi K, Nikpour Y, Fallah-Mehrjardi M (2012). Application of sulfur microparticles for solid-phase extraction of polycyclic aromatic hydrocarbons from sea water and wastewater samples. Anal Chem Acta.

[7] Rodenburg LA (2006). Mass Balances on Selected Polycyclic Aromatic Hydrocarbons (PAHs) in the NY/NJ Harbor Estuary.

[8] WHO (1998). Polycyclic Aromatic Hydrocarbons, Selected Non-Heterocyclic (EHC 202, 1998).

[9] WHO (1998). Guidelines for Drinking-Water Quality [electronic resource]: incorporating first addendum. Vol. 1, Recommendations.-3rd ed.

[10] WHO (1998). Guidelines for Drinking-Water Quality. 2nd Edition, Vol. 2, health criteria and other supporting information-Addendum.

[11] Draboval L, Pulkrabova J, Kalachova K, Hradecky J, Suchanova M, Tomaniova M, Kocourek V, Hajslova J (2011). Novel approaches to determination of PAHs and halogenated POPs in canned fish. Czech J Food Sci.

[12] Suchanova M, Hajslova J, Tomaniov M, Kocourek V, Babicka L (2008). Polycyclic aromatic hydrocarbons in smoked cheese. J Sci Food Agric.

[13] WHO (2010). International Agency for Research on Cancer (IARC) Monographs on the Evaluation of Carcinogenic Risks to Humans, Some Non-heterocyclic Polycyclic Aromatic Hydrocarbons and Some Related Exposures.

[14] Mastrangelo G, Fadda E, Marzia V (1996). Polycyclic aromatic hydrocarbons and cancer in man. Environ Health Perspect.

[15] Watson DH (2004). Pesticide Veterinary and other Residues in Food. Woodhead Publishing Limited and CRC Press, England.

[16] Food Standards Agency (2012). Polycyclic Aromatic Hydrocarbons in Cereals, Cereal Products, Vegetables, Vegetable Products and Traditionally Smoked Foods. No. 01/12. https://www.food.gov.uk/science/research/chemical-safety-research/env-cont/c02090.

[17] Prieto A, Schrader S, Moeder M (2010). Determination of organic priority pollutants and emerging compounds in wastewater and snow samples using multiresidue protocols on the basis of microextraction by packed sorbents coupled to large volume injection gas chromatography–mass spectrometry analysis. J Chromatogr A.

[18] Pena MT, Casais MC, Mejuto MC, Cela R (2009). Development of an ionic liquid based dispersive liquid–liquid microextraction method for the analysis of polycyclic aromatic hydrocarbons in water samples. J Chromatogr A.

[19] Ma X, Huang M, Li Z, Wu J (2011). Hollow fiber supported liquid-phase microextraction using ionic liquid as extractant for preconcentration of benzene, toluene, ethylbenzene and xylenes from water sample with gas chromatography-hydrogen flame ionization detection. J Hazardous Materials.

[20] Sarafraz-Yazdi A, Amiri A (2010). Liquid-phase microextraction. Trends Anal Chem.

[21] Dasgupta S, Banerjeea K, Utturea S, Kusarie P, Wagha S, Dhumalc K, Kolekard S, Adsulea PG (2011). Extraction of pesticides, dioxin-like PCBs and PAHs in water based commodities using liquid–liquid microextraction and analysis by gas chromatography–mass spectrometry. J Chromatogr A.

[22] Rial-Otero R, Cancho-Grande B, Simal-Gandara J (2003). Multiresidue method for fourteen fungicides in white grapes by liquid-liquid and solid phase extraction followed by liquid chromatography-diode array detection. J Chromatogr A.

[23] Pose-Juan E, Cancho-Grande B, Rial-Otero R, Simal-Gandara J (2006). The dissipation rates of cyprodinil, fludioxonil, procymidone and vinclozoline during storage of grape juice. Food Control.

[24] Leong MI, Chang CC, Fuh MR, Huang SD (2010). Low toxic dispersive liquid–liquid microextraction using halosolvents for extraction of polycyclic aromatic hydrocarbons in water samples. J Chromatogr A.

[25] Hodgeson JW Determination of Polycyclic Aromatic Hydrocarbons in Drinking Water by Liquid–Liquid Extraction and HPLC with Coupled Ultraviolet and Fluorescence Detection. U.S. EPA (1990) Method 550.

[26] Khalili Zanjani MR, Yamini Y, Shariati S, Jönsson JA (2007). new liquid-phase microextraction method based on solidification of floating organic drop. Anal Chim Acta.

[27] Galán-Cano F, Bernabé-Zafón V, Lucena R, Cárdenas S, Herrero-Martínez JM, Ramis-Ramos G, Valcárcel M (2011). Sensitive determination of polycyclic aromatic hydrocarbons in water samples using monolithic capillary solid-phase extraction and on-line thermal desorption prior to gas chromatography–mass spectrometry. J Chromatogr A.

[28] Xu H, Ding Z, Lv L, Song D, Feng YQ (2009). A novel dispersive liquid–liquid microextraction based on solidification of floating organic droplet method for determination of polycyclic aromatic hydrocarbons in aqueous samples. Anal Chim Acta.

[29] López-Blanco MC, Cancho-Grande B, Simal-Gándara J (2002). Comparison of solid-phase extraction and solid-phase microextraction for carbofuran in water analyzed by high-performance liquid chromatography–photodiode-array detection. J Chromatogr A.

[30] García-Falcón MS, Pérez-Lamela M, Simal-Gándara J (2004). Comparison of strategies for extraction of high molecular weight polycyclic aromatic hydrocarbons from drinking waters. J Agric Food Chem.

[31] López-Blanco C, Gómez-Alvarez S, Rey-Garrote M, Cancho-Grande B, Simal-Gándara J (2006). Determination of pesticides by solid phase extraction followed by gas chromatography with nitrogen-phosphorous detection in natural water and comparison with solvent drop microextraction. Anal Bioanal Chem.

[32] Liu Y, Li HF, Zhang JH, Maeda T, Lin JM (2010). Triacontyl modified silica gel as a sorbent for the preconcentration of polycyclic aromatic hydrocarbons in aqueous samples prior to gas chromatographic-mass spectrometry determination. Chinese Chem Lett.

[33] Guo L, Lee HK (2011). Low-density solvent-based solvent demulsification dispersive liquid–liquid microextraction for the fast determination of trace levels of sixteen priority polycyclic aromatic hydrocarbons in environmental water samples. J Chromatogr A.

[34] He Y, Lee HK (1997). Liquid-phase microextraction in a single drop of organic solvent by using a conventional microsyringe. Anal Chem.

[35] Hou L, Lee HK (2002). Application of static and dynamic liquid-phase microextraction in the determination of polycyclic aromatic hydrocarbons. J Chromatogr A.

[36] Wu H, Wang X, Liu B, Lu J, Du B, Zhang L, Ji J, Yue Q, Han B (2010). Flow injection solid-phase extraction using multi-walled carbon nanotubes packed micro-column for the determination of polycyclic aromatic hydrocarbons in water by gas chromatography–mass spectrometry. J Chromatogr A.

[37] Leong MI, Huang SD (2008). Dispersive liquid–liquid microextraction method based on solidification of floating organic drop combined with gas chromatography with electron-capture or mass spectrometry detection. J Chromatogr A.

[38] Rezaee M, Assadi Y, Milani Hosseini MR, Aghaee E, Ahmadi F, Berijani S (2006). Determination of organic compounds in water using dispersive liquid–liquid microextraction. J Chromatogr A.

[39] Farahani H, Norouzi P, Dinarvand R, Ganjali MR (2007). Development of dispersive liquid–liquid microextraction combined with gas chromatography–mass spectrometry as a simple, rapid and highly sensitive method for the determination of phthalate esters in water samples. J Chromatogr A.

[40] Rahnama Kozani R, Assadi Y, Shemirani F, Milani Hosseini MR, Jamali MR (2007). Determination of trihalomethanes in drinking water by dispersive liquid–liquid microextraction then gas chromatography with electron-capture detection. J Chromatogr A.

[41] Hung K, Chen BH, Yu LE (2007). Cloud-point extraction of selected polycyclic aromatic hydrocarbons by nonionic surfactants. Sep Purif Technol.

[42] Aubert C, Baumann S, Arguel H (2005). Optimization of the analysis of flavor volatile compounds by liquid-liquid microextraction (LLME). Application to the aroma analysis of melons, peaches, grapes, strawberries, and tomatoes. Agric Food Chem.

[43] He Y, Vargas A, Kang YJ (2007). Headspace liquid-phase microextraction of methamphetamine and amphetamine in urine by an aqueous drop. Anal Chim Acta.

[44] Saleh A, Yamini Y, Faraji M, Rezaee M, Ghambarian M (2009). Ultrasound-assisted emulsification microextraction method based on applying low density organic solvents followed by gas chromatography analysis for the determination of polycyclic aromatic hydrocarbons in water samples. J Chromatogr A.

[45] Merino F, Rubio S, Pérez-Bendito D (2002). Acid– induced cloud point extraction and preconcentration of polycyclic aromatic hydrocarbons from environmental solid samples. J Chromatogr A.

[46] Pena-Pereira F, Lavilla I, Bendicho C (2009). Miniaturized preconcentration methods based on liquid-liquid extraction and their application in inorganic ultratrace analysis and speciation: A review. Spectrochim Acta B.

[47] (2011). Commission Regulation (EC) No 333/2007 laying down the methods of sampling and analysis for the official control of the levels of lead, cadmium, mercury, inorganic tin, 3-MCPD and benzo(a)pyrene in foodstuffs.

[48] Al-Mudhaf HF, Alsharifi FA, Abu-Shady AS (2009). A survey of organic contaminants in household and bottled drinking waters in Kuwait. Sci Total Environ.

[49] Pena MT, Casais MC, Mejuto MC, Cela R Development of an ionic liquid based dispersive liquid–liquid microextraction method for the analysis of polycyclic aromatic hydrocarbons in water samples. J Chromatogr A.

[50] Guart A, Calabuig I, Lacorte S, Borrell A (2014). Environ continental bottled water assessment by stir bar sorptive extraction followed by gas chromatography-tandem mass spectrometry (SBSE-GC-MS/MS). Sci Pollut Res.

[51] Guler C (2007). Evaluation of maximum contaminant levels in Turkish bottled drinking waters utilizing parameters reported on manufacturer’s labeling and government-issued production licenses. J Food Comp Anal.

